# Scrub typhus islands in the Taiwan area and the association between scrub typhus disease and forest land use and farmer population density: geographically weighted regression

**DOI:** 10.1186/1471-2334-13-191

**Published:** 2013-04-29

**Authors:** Pui-Jen Tsai, Hsi-Chyi Yeh

**Affiliations:** 1 Center for General Education, Aletheia University, New Taipei 25103, Republic of China

**Keywords:** Scrub typhus, Climate, Farm worker population density, Forest-land uses, Geographically weighted regression

## Abstract

**Background:**

The Taiwan area comprises the main island of Taiwan and several small islands located off the coast of the Southern China. The eastern two-thirds of Taiwan are characterized by rugged mountains covered with tropical and subtropical vegetation. The western region of Taiwan is characterized by flat or gently rolling plains. Geographically, the Taiwan area is diverse in ecology and environment, although scrub typhus threatens local human populations. In this study, we investigate the effects of seasonal and meteorological factors on the incidence of scrub typhus infection among 10 local climate regions. The correlation between the spatial distribution of scrub typhus and cultivated forests in Taiwan, as well as the relationship between scrub typhus incidence and the population density of farm workers is examined.

**Methods:**

We applied Pearson’s product moment correlation to calculate the correlation between the incidence of scrub typhus and meteorological factors among 10 local climate regions. We used the geographically weighted regression (GWR) method, a type of spatial regression that generates parameters disaggregated by the spatial units of analysis, to detail and map each regression point for the response variables of the standardized incidence ratio (SIR)-district scrub typhus. We also applied the GWR to examine the explanatory variables of types of forest-land use and farm worker density in Taiwan in 2005.

**Results:**

In the Taiwan Area, scrub typhus endemic areas are located in the southeastern regions and mountainous townships of Taiwan, as well as the Pescadore, Kinmen, and Matou Islands. Among these islands and low-incidence areas in the central western and southwestern regions of Taiwan, we observed a significant correlation between scrub typhus incidence and surface temperature. No similar significant correlation was found in the endemic areas (e.g., the southeastern region and the mountainous area of Taiwan). Precipitation correlates positively with scrub typhus incidence in 3 local climate regions (i.e., Taiwan’s central western and southwestern regions, and the Kinmen Islands). Relative humidity correlates positively with incidence in Southwestern Taiwan and the Kinmen Islands. The number of wet days correlates positively with incidence in Southwestern Taiwan. The duration of sunshine correlates positively with incidence in Central Western Taiwan, as well as the Kinmen and Matou Islands. In addition, the 10 local climatic regions can be classified into the following 3 groups, based on the warm-cold seasonal fluctuations in scrub typhus incidence: (a) Type 1, evident in 5 local climate regions (Taiwan’s northern, northwestern, northeastern, and southeastern regions, as well as the mountainous area); (b) Type 2 (Taiwan’s central western and southwestern regions, and the Pescadore Islands); and (c) Type 3 (the Kinmen and Matou Islands). In the GWR models, the response variable of the SIR-district scrub typhus has a statistically significantly positive association with 2 explanatory variables (farm worker population density and timber management). In addition, other explanatory variables (recreational forests, natural reserves, and “other purpose” areas) show positive or negative signs for parameter estimates in various locations in Taiwan. Negative signs of parameter estimates occurred only for the explanatory variables of national protectorates, plantations, and clear-cut areas.

**Conclusion:**

The results of this study show that scrub typhus in Taiwan can be classified into 3 types. Type 1 exhibits no climatic effect, whereas the incidence of Type 2 correlates positively with higher temperatures during the warm season, and the incidence of Type 3 correlates positively with higher surface temperatures and longer hours of sunshine. The results also show that in the mountainous township areas of Taiwan’s central and southern regions, as well as in Southeastern Taiwan, higher SIR values for scrub typhus are associated with the following variables: farm worker population density, timber management, and area type (i.e., recreational forest, natural reserve, or other purpose).

## Background

Scrub typhus is an acute infectious disease in humans that is caused by the intracellular bacteria *Orientia tsutsugamushi* (*Rickettsia tsutsugamushi*, OT), which belongs to the Rickettsiaceae family [1-3]. Scrub typhus is prevalent within a 13,000,000-km^2^ area of the Asia-Pacific rim, extending from Northern Japan and Eastern Russia to Northern Australia, and to Pakistan and Afghanistan in the west [4-6]. OT infection is transmitted to people and rodents by various species of infected trombiculid mites that feed on lymph and tissue fluid. Such mites are approximately 0.2 to 0.4 mm in length, and have a 4-stage life cycle (egg, larva, nymph, and adult). The larval stage is the only parasitic stage when the pathogen is transmitted to people and other vertebrates. In regions where scrub typhus is a constant threat, a natural cycle of OT transmission occurs through transovarial transfer from mite larvae to small mammals, such as field mice or rats, and people are incidental hosts [5,7].

The seasonal occurrence of scrub typhus varies according to the climates in different countries, and the disease occurs more commonly during rainy seasons. Forest clearings, riverbanks, and grassy regions provide optimal conditions for mites [1,5,7-10]. The relationship between scrub typhus incidence and climate type reflects the behavior and population densities of trombiculidae within various environments [5].

Taiwan is situated near mainland China. The Tropic of Cancer divides the island into the following 2 climatic zones: (a) the tropical monsoon climate in the south; and (b) the subtropical monsoon climate in the north. The latitude, topography, ocean currents, and monsoons of Taiwan contribute to the island’s high temperatures, humidity, and rainfall, as well as tropical cyclones during summer. According to the Köppen–Geiger climate classification system, the 4 primary climate types in Taiwan are: (a) the monsoon and trade-wind coastal climate in the south; (b) the mild and humid climate in the north; (c) the wet-dry tropical climate in the west; and (d) the temperate rainy climate with dry winters in mountainous areas [11]. Previous studies have reported a higher risk for scrub typhus infection in the Matuo (Lienchiang County), Kinmen (Kinmen County), and Pescadore Islands (Penghu County), with endemic clusters in Southeastern Taiwan (i.e., plain townships within Hualien and Taitung Counties) and Taiwan’s mountainous township areas [12-15]. The Taiwan area, which comprises the main island of Taiwan and several small islands (i.e., the Pescadore, Kinmen, Matuo, Little Liuchiu, Green, and Orchid Islands) is geographically isolated and characterized by various climate types and ecologies. However, there is little understanding on the influence of these factors on scrub typhus incidence. Specifically, research on the effects of local climates on scrub typhus incidence, as well as the effects of the geographical distribution of forest-land use and farm worker population density on the rate of infection, is rare.

In this study, we investigated the effects of meteorological factors within local climatic regions on the seasonal incidence of scrub typhus. We applied geographically weighted regression (GWR), which is a spatial statistical tool, to study the spatial distribution of standardized incidence ratio (SIR)-district scrub typhus. We also examined the correlation between SIR and 8 environmental and socioeconomic factors among Taiwan’s population during 2005.

## Methods

### Study area and local climate regions

**Figure 1:**
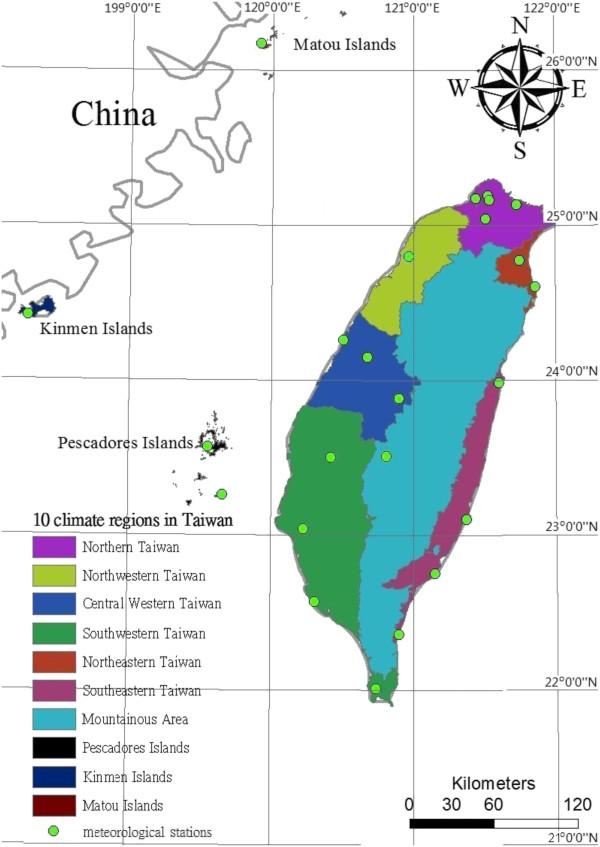
Ten climatic regions and 24 local meteorological stations in Taiwan.

We focused our analysis on the main island of Taiwan and specific islands in the region (the Pescadore, Kinmen, and Matou Islands). Smaller, more isolated islands, such as the Little Liuchiu, Green, and Orchid Islands, were excluded. We collected data recorded between 1897 and 2008 at 25 traditional surface stations. Taiwan’s Central Weather Bureau (CWB) divided the regional climate and associated geographical areas into the following 6 regions (4 metropolitan areas in Northern, Central, Southern, and Eastern Taiwan, and 2 mountainous regions in Northern and Central Taiwan) [16]. Because the regional climate differs markedly between the northeastern and southeastern regions of Taiwan, for the purpose of this study, we divided the geographical area of Eastern Taiwan into 2 regions, resulting in 7 categories. We subsequently added an additional 3 neighboring islands for a total of 10 geographical regions in Taiwan (Figure 1). For our analysis, we used obtained meteorological data from 24 meteorological stations that were collected from 2002 to 2011. We also obtained data on meteorological variables from the CWB, based on monthly observations (e.g., surface temperature, precipitation, relative humidity, atmospheric pressure, number of wet days, and hours of sunshine) [17]. Table 1 shows a summary of the mean, median, and range of meteorological factors for the 10 local climate regions of this study.

**Table 1:** The 10 local climatic regions and their monthly meteorological factors in Taiwan (2002–2011)

**Local climate regions**	**Meteorological factors**	**Mean**	**Median**	**Range**
Northern Taiwan	surface temperature^b^ (°C)	20.9	21.3	11.3-28.2
	precipitation^c^ (mm)	274	225	31-1306
	relative humidity^b^ (%)	80.7	80.8	69.5-89.8
	atmospheric pressure^b^ (hecto-Pascal)	981	982	971-994
	wet days^d^ (day)	14.5	14.6	3.8-26.4
	sunshine duration^c^ (hour)	115	111	13-224
Northwestern Taiwan	surface temperature^b^ (°C)	22.8	23.2	13.3-30
	precipitation^c^ (mm)	140	91	0-712
	relative humidity^b^ (%)	76.3	76	64-86
	atmospheric pressure^b^ (hecto-Pascal)	1010	1011	1000-1019
	wet days^d^ (day)	9.7	9.5	1-19
	sunshine duration^c^ (hour)	156	150	29-296
Central western Taiwan	surface temperature^b^ (°C)	22	22.9	13.7-27.8
	precipitation^c^ (mm)	155	85	0-935
	relative humidity^b^ (%)	77.6	77.7	70-85
	atmospheric pressure^b^ (hecto-Pascal)	971	972	963-978
	wet days^d^ (day)	9.4	8.8	0.3-21
	sunshine duration^c^ (hour)	163	166	85-245
Southwestern Taiwan	surface temperature^b^ (°C)	24.7	25.7	16.7-30.1
	precipitation^c^ (mm)	154	62	0-1091
	relative humidity^b^ (%)	75.3	75	65.3-83.3
	atmospheric pressure^b^ (hecto-Pascal)	1010	1011	1002-1018
	wet days^d^ (day)	7.5	5.9	0-21.8
	sunshine duration^c^ (hour)	186	184	95-285
Northeastern Taiwan	surface temperature^b^ (°C)	22.8	23.1	13.9-30
	precipitation^c^ (mm)	277	222	2-1381
	relative humidity^b^ (%)	78.7	79	68.5-86.5
	atmospheric pressure^b^ (hecto-Pascal)	1012	1013	1001-1021
	wet days^d^ (day)	16	15.8	1-28
	sunshine duration^c^ (hour)	123	106	11-314
Southeastern Taiwan	surface temperature^b^ (°C)	24.3	24.7	17.6-30
	precipitation^c^ (mm)	172	84	8-859
	relative humidity^b^ (%)	75.1	75.6	64.8-82
	atmospheric pressure^b^ (hecto-Pascal)	1011	1012	1001-1019
	wet days^d^ (day)	11.8	11.8	2.5-20.3
	sunshine duration^c^ (hour)	142	132	24-323
Mountainous Area	surface temperature^b^ (°C)	11.4	12.2	4.3-15.5
	precipitation^c^ (mm)	361	160	0-3346
	relative humidity^b^ (%)	87.4	88.5	71-98
	atmospheric pressure^b^ (hecto-Pascal)	764	765	761-767
	wet days^d^ (day)	13	12.5	0-28
	sunshine duration^c^ (hour)	124	121	52-216
Pescadore Islands	surface temperature^b^ (°C)	23.8	24.5	14-29.7
	precipitation^c^ (mm)	91	44	0-586
	relative humidity^b^ (%)	79.3	80	68-86.5
	atmospheric pressure^b^ (hecto-Pascal)	1010	1010	1001-1018
	wet days^d^ (day)	5.6	5.5	0-15
	sunshine duration^c^ (hour)	173	176	35-345
Kinmen Islands^a^	surface temperature^b^ (°C)	20.9	21.5	10.6-29.1
	precipitation^c^ (mm)	90	68	0-487
	relative humidity^b^ (%)	77.8	77	61-93
	atmospheric pressure^b^ (hecto-Pascal)	1011	1012	1000-1021
	wet days^d^ (day)	7.8	7	0-20
	sunshine duration^c^ (hour)	158	156	37-292
Matou Islands^a^	surface temperature^b^ (°C)	19	19.7	7.1-28.1
	precipitation^c^ (mm)	94	67	0-344
	relative humidity^b^ (%)	84.2	85	67-92
	atmospheric pressure^b^ (hecto-Pascal)	1004	1006	994-1016
	wet days^d^ (day)	9.5	9	0-19
	sunshine duration^c^ (hour)	145	139	26-325

^a^ indicates that available data from 2004 to 2011 was used for this variable.

^b^The averaged value was calculated for each month.

^c^The value was accumulated for every day in a month.

^d^The measured value all day long was larger than 0.1 mm (defined as one wet day), and dry otherwise.

### Incidence of scrub typhus in people

Data for confirmed cases of scrub typhus were obtained from the Notifiable Infectious Diseases Statistics System and Infectious Diseases Database at the Taiwanese Center for Disease Control (CDC) [18]. Because scrub typhus is a notifiable disease, blood samples from patients with suspected scrub typhus were collected and sent to the CDC for laboratory confirmation. The samples were labeled positive for scrub typhus based on a positive real-time polymerase chain reaction test, or a 4-fold increase in OT-specific immunoglobulin M or immunoglobulin G antibody of paired sera by using an indirect immunofluorescence assay technique. Data were obtained for the years 2002 through 2011. Ethical approval for this study was unnecessary because the data are public domain.

### Data management

Data on the population density of farm workers and the use of forest land (e.g., timber management, recreational forests, national protectorate, natural reserves, plantations, clear-cut areas, and other purposes) were obtained from the 2005 Agricultural, Forestry, Fishery, and Husbandry census [19], which also included the socioeconomic and environmental status represented by variables used in our analysis. The calculated SIR of scrub typhus for each township was subsequently used as the response variable in the GWR model. The GWR model used the following explanatory variables: (a) percentage of farm labor; and (b) land share ratio of timber management, recreational forests, national protectorates, natural reserves, plantations, clear-cut areas, and other purposes.

We used the statistical software package SPSS (v.12) to calculate the Pearson’s product–moment correlations (*r*), and employed ArcMap (v.9.3) to map the GWR.

### General statistics

We applied the chi-square goodness-of-fit and Fisher exact tests [20] to analyze the seasonal variation within and among the 10 local climatic regions. The strength of the relationships between scrub typhus incidence and meteorological variables across the 10 regions were assessed based on Pearson’s *r*.

### Geographically weighted regression

The GWR method is a spatial statistical tool that generates parameters disaggregated by the spatial units of analysis. We considered analyzing the contiguity-based spatial units (e.g., 349 administrative government areas on the main island of Taiwan) by using the GWR method. However, this method is unsuitable for examining isolated regions (e.g., the Pescadore, Kinmen, and Matou Islands). The GWR model is an extension of the traditional standard regression framework that estimates local rather than global parameters [21]. It is a type of local statistic that produces a set of local parameter estimates showing the spatial variation of relationships. Thus, the spatial pattern of local estimates can be examined to elucidate potentially ambiguous causes of observed differences [22]. Conversely, traditional regression methods, such as the ordinary least squares (OLS) method, are global statistical tools that assume the spatial constancy of a particular relationship (i.e., a parameter is assumed to remain constant across an entire area).

An OLS model can be defined as follows:

$$y=\beta_{0}+\sum_{i=1}^{y}\beta_{i}X_{i}+\epsilon$$

where *y* is the response variable, *β*_0_ is the intercept, *β*_*i*_ is the parameter estimate (coefficient) for explanatory variable *x*_*i*_, *p* is the number of explanatory variables, and *ϵ* is the error term.

The GWR model allows local rather than global parameters to be estimated for the study area. Thus, the GWR model rewrites the OLS model as follows:

(1) $$y_{j}=\beta_{0}\left(u_{j},v_{j}\right)+\sum_{i=1}^{y}\beta_{i}\left(u_{j},v_{j}\right)x_{\mathrm{ij}}+\epsilon_{j}$$

where *u*_*j*_ and *v*_*j*_ are the coordinates for each location *j*, *β*_0_ (*u*_*j*_,*v*_*j*_) is the intercept for location *j*, and *β*_*i*_ (*u*_*j*_,*v*_*j*_) is the local parameter estimate for explanatory variable *x*_*i*_ at location *j*. The weight assigned to each observation is based on a distance decay function centered on observation *i*.

The estimator for the GWR model is similar to the weighted least squares global model. However, in the GWR model, the weights are determined by location *u*, which is relative to the observations in the data set, and consequently changes for each location. The estimator is expressed as follows:

(2) $$\hat{\beta }\left(u\right)={\left(X^{T}W\left(u\right)X\right)}^{-1}X^{T}W\left(u\right)y$$

where *W*(*u*) is the square matrix of weights relative to position *u*. A specific location can be indexed (*u*_*j*_,*v*_*j*_) in the study area. The geographically weighted variance-covariance matrix is represented by *X*^*T*^*W*(*u*)*X*, and *y* is the vector of the value of the response variable. The *W*(*u*) matrix contains the geographical weights in its leading diagonal elements, and zero in its off-diagonal elements.

(3) $$\left[\begin{array}{cccc} w_{1}\left(u\right) & 0 & 0 & 0\\ 0 & w_{2}\left(u\right) & 0 & 0\\ 0 & 0 & \ldots & 0\\ 0 & 0 & 0 & w_{n}\left(u\right) \end{array}\right]$$

In the area that this study was conducted, the sample points produced by the polygon centroids were clustered rather than placed regularly. A convenient method for implementing an adaptive bandwidth specification is to select a kernel that allows an identical number of sample points for estimations. The weight can subsequently be calculated using the selected kernel and by setting the value for any observation with a distance that exceeds the bandwidth to zero. The bisquare function is expressed as follows:

(4) wiuj,vj=1−diuj,vjh22

where *w*_*i*_(*u*_*j*_,*v*_*j*_) is zero when *d*_*i*_(*u*_*j*_,*v*_*j*_) is greater than *h*. The variable *h* represents the bandwidth quantity, which is a near-Gaussian function with the useful property of the weight being zero at a finite distance.

In this study, we selected the bandwidth by minimizing the Akaike information criterion (AIC) score by using the following equation:

(5) $$\mathrm{AI}C_{c}=2n\log_{e}\left(\hat{\sigma }\right)+n\log_{e}\left(2\pi \right)+n\left\{\frac{n+\mathrm{tr}\left(S\right)}{n-2-\mathrm{tr}\left(S\right)}\right\}$$

where *tr*(*S*) is the trace of the hat matrix. The AIC method is advantageous because it considers the possible variation in degrees of freedom among models centered on various observations. We determined the optimal bandwidth by minimizing the adjusted AIC, as detailed by Fotheringham et al. (2002) [22]. GWR models produce a set of local regression results, including local parameter estimates and local residuals, which can be mapped to show their spatial variability.

The Benjamini-Hochberg (B-H) procedure modifies the significance level for each test consistently. We applied this procedure to control the false discovery rate in multiple comparisons and to determine the significance of parameter estimates obtained using the GWR model. Thissen et al. (2002) proposed a simple method for calculating the B-H procedure false discovery rate by using Microsoft Excel [23]. The B-H approach controls the FDR by sequentially comparing the observed p values for each family of multiple test statistics (from largest to smallest) with a list of computed B-H critical values [*pB-H*(*i*)]. The critical value on the list is determined for each test statistic, and is indexed by *i* through linear interpolation between α/2 (for the largest observed p value) to (α/2)/*m*, where *m* is the family size (for the smallest of the p values). Because the final value is the Bonferroni critical value, the reason for the gain in the power of B-H relative to the Bonferroni approach is as follows: the B-H approach compares only the smallest of the *m* observed p values with the Bonferroni critical value. All other p values are calculated using less stringent criteria. The local parameter is considered significant if the p value is less than the B-H critical value; otherwise, the parameter is considered non-significant [23].

## Results

### Correlation between cases of scrub typhus and meteorological variables

Table 2 shows a summary of correlations between scrub typhus incidence and meteorological variables from 2002 to 2011. The results were statistically non-significant for 4 of the local climate regions of the main island of Taiwan (the northern, northeastern, southeastern, and mountainous area of Taiwan). In Northwestern Taiwan, incidence rates correlate negatively with relative humidity. In Central Western Taiwan, OT incidence correlates positively with surface temperature, precipitation, and the duration of sunshine, although the correlation with atmospheric pressure is negative. In Southwestern Taiwan, OT incidence correlates positively with surface temperature, precipitation, relative humidity, and wet days, whereas the correlation with atmospheric pressure is negative. In the Pescadore Islands, OT incidence correlates positively with surface temperature, whereas the correlation with atmospheric pressure is negative. In the Kinmen Islands, OT incidence correlates positively with surface temperature, precipitation, relative humidity, and the duration of sunshine, whereas the correlation with atmospheric pressure is negative. In the Matou Islands, OT incidence correlates positively with surface temperature and sunshine duration, although the correlation with atmospheric pressure is negative.

**Table 2:** Correlations between the monthly incidences and meteorological factors for scrub typhus in 10 local climate regions of Taiwan (2002–2011)

**Local climate regions**	**Surface temperature**^**b**^	**Precipitation**^**c**^	**Relative humidity**^**b**^	**Atmospheric pressure**^**b**^	**Wet days**^**d**^	**Sunshine duration**^**c**^
	**(°C)**	**(mm)**	**(%)**	**(hBa)**	**(>0.1 mm/day)**	**(hour)**
Northern Taiwan	0.182	−0.02	−0.09	−0.14	−0.12	0.213
Northwestern Taiwan	0.016	−0.09	−0.243*	0.00	−0.14	0.16
Central Western Taiwan	0.475*	0.249*	0.00	−0.450*	0.12	0.400*
Southwestern Taiwan	0.422*	0.279*	0.276*	−0.411*	0.381*	0.16
Northeastern Taiwan	−0.176	−0.07	−0.10	0.14	0.00	−0.08
Southeastern Taiwan	0.202	0.16	−0.08	−0.09	0.01	0.18
Mountainous Area	0.187	0.14	0.07	−0.16	0.12	0.04
Pescadore Islands	0.370*	0.15	0.08	−0.316*	0.15	0.188
Kinmen Islands^a^	0.623*	0.354*	0.567*	−0.630*	0.11	0.473*
Matou Islands^a^	0.583*	0.00	0.18	−0.535*	−0.227	0.592*

* *p* < 0.01.

^a^ indicates that available data from 2004 to 2011 was used for this variable.

^b^The averaged value was calculated for each month.

^c^The value was accumulated for every day in a month.

^d^The measured value all day long was larger than 0.1 mm (defined as one wet day), and dry otherwise.

Surface temperature correlates positively with scrub typhus incidence in 5 local climate regions (Central Western and Southwestern Taiwan, as well as the Pescadore, Kinmen, and Matou Islands), although the correlation between OT incidence and atmospheric pressure in these regions is negative. Precipitation correlates positively with OT incidence in 3 local climate regions (Central Western and Southwestern Taiwan, and the Kinmen Islands). Relative humidity correlates positively with OT incidence in 2 regions (Southwestern Taiwan and the Kinmen Islands), whereas the correlation with OT incidence is negative in Northwestern Taiwan. The number of wet days correlates positively with OT incidence only in the southwestern region of Taiwan. The duration of sunshine correlates positively with OT incidence in 3 regions (Central Western Taiwan, and the Kinmen and Matou Islands).

### Seasonal variations in scrub typhus incidence

**Figure 2:**
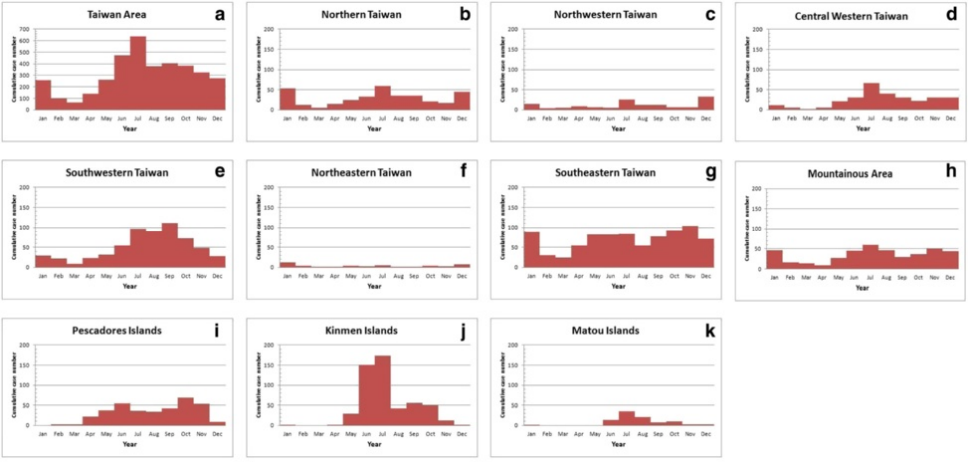
**Seasonal incidence of scrub typhus in the Taiwan area and 10 local climate regions (2002–2011): (a) the Taiwan area, (b) Northern Taiwan, (c) Northwestern Taiwan, (d) Central Western Taiwan, (e) Southwestern Taiwan, (f) Northeastern Taiwan, (g) Southeastern Taiwan, (h) the mountainous area, (i) Pescadore Islands, (j) Kinmen Islands, and (k) Matou Islands.**

Table 3 shows a summary of the seasonal variations in scrub typhus infection from 2002 to 2011 in the mentioned 10 local climate regions. In the Taiwan area, the seasonal variation in the incidence of scrub typhus was significantly higher during the warm season than during the cold season (χ^2^ = 517.4, *P* < .01). Peaks occurred in June (472 cases) and July (638 cases) throughout the Taiwan area (Figure 2a). For Northern Taiwan, the incidence rate during the warm season was significantly higher than during the cold season (χ^2^ = 10.5, *P* < .01). Peaks occurred in January (23 cases) and July (59 cases) in Northern Taiwan (Figure 2b). In Northwestern Taiwan, the seasonal variation was similar to that of the cold season (χ^2^ = 0.18, *P* = .67). Peaks occurred in July (26 cases) and December (33 cases) in Northwestern Taiwan (Figure 2c). In Central Western Taiwan, the incidence in the warm season was significantly higher than that in the cold season (χ^2^ = 54, *P* < .01). A peak occurred in July (66 cases) in Central Western Taiwan (Figure 2d). In Southwestern Taiwan, the results show that the incidence was significantly higher during the warm season compared to the cold season (χ^2^ = 143, *P* < .01). Peaks occurred in July (96 cases), August (91 cases), and September (111 cases) in Southwestern Taiwan (Figure 2e). In Northeastern Taiwan, the incidence in the warm season was similar to that of the cold season (χ^2^ = 2, *P* = .16). Peaks occurred in January (12 cases) and December (8 cases) in Northeastern Taiwan (Figure 2f). In Southeastern Taiwan, the incidence during the warm season was significantly higher than during the cold season (χ^2^ = 11.8, *P* < .01). A peak occurred in November (104 cases, Figure 2g). In the mountainous area, the incidence in the warm season was significantly higher than during the cold season (χ^2^ = 10, *P* < .01). A peak appeared in July (59 cases) in the mountainous area (Figure 2h).

**Table 3:** Seasonal differences of scrub typhus infection from 2002 to 2011 in 10 local climate regions of Taiwan

		**Cases (%)**					
**Local climate regions**	**Total**	**Spring**	**Summer**	**Autumn**	**Winter**	**Warm**	**Cold**
Taiwan Area	3697	468 (12.7)	1488 (40.2)	1115 (30.2)	626 (16.9)	2540 (68.7)	1157 (31.3)
Northern Taiwan	355	45 (12.7)	127 (35.8)	73 (20.6)	110 (31)	208 (58.6)	147 (41.4)
Northwestern Taiwan	139	20 (14.4)	43 (30.9)	24 (17.3)	52 (37.4)	67 (48.2)	72 (51.8)
Central Western Taiwan	294	28 (9.5)	137 (46.6)	82 (27.9)	47 (16)	210 (71.4)	84 (28.6)
Southwestern Taiwan	617	64 (10.4)	241 (39.1)	233 (37.8)	79 (12.8)	457 (74.1)	160 (25.9)
Northeastern Taiwan	50	7 (14)	10 (20)	9 (18)	24 (48)	20 (40)	30 (60)
Southeastern Taiwan	844	160 (19)	220 (26.1)	274 (32.5)	190 (22.5)	472 (55.9)	372 (44.1)
Mountainous Area	423	50 (11.8)	150 (35.5)	117 (27.7)	106 (25.1)	244 (57.7)	179 (42.3)
Pescadore Islands	364	63 (17.3)	125 (34.3)	164 (45.1)	12 (3.3)	274 (75.3)	90 (24.7)
Kinmen Islands	516	31 (6)	365 (70.7)	118 (22.9)	2 (0.4)	500 (96.9)	16 (3.1)
Matou Islands	95	0 (0)	70 (73.7)	21 (22.1)	4 (4.2)	88 (92.6)	7 (7.4)

Four seasons were defined: spring (from March to May), summer (from June to August), autumn (from September to November), and winter (from December to February).

Warm-cold seasons were defined: warm season (from May to October), and cold season (from November to April).

In the Pescadore Islands, the incidence was significantly higher during the warm months than during the cold months (χ^2^ = 93, *P* < .01). Peaks occurred in June (55 cases) and October (69 cases) in the Pescadore Islands (Figure 2i). In the Kinmen Islands, the incidence during the warm season was significantly higher than that of the cold season (χ^2^ = 454, *P* < .01). Peaks occurred in June (150 cases) and July (173 cases) in the Kinmen Islands (Figure 2j). In the Matou Islands, the incidence in the warm season was significantly higher than during the cold season (χ^2^ = 69.1, *P* < .01). Peaks occurred in July (35 cases) and August (21 cases) in the Matou Islands (Figure 2k).

### Comparison of seasonal variation among the 10 regions

Table 4 shows a summary of the warm-cold seasonal comparisons in scrub typhus incidences among the 10 local climate regions. A p value less than .01 is statistically significant, and such regions were considered to exhibit divergent seasonal patterns. If a p value was greater than.01, then the compared regions were considered to exhibit similar seasonal patterns.

**Table 4:** **Matric comparisons between the seasonal patterns with warm-cold cycles among 10 climate regions of Taiwan (2002–2011)**^**$**^

**Climate regions of Taiwan**	**Northern Taiwan**	**Northwestern Taiwan**	**Central Western Taiwan**	**Southwestern Taiwan**	**Northeastern Taiwan**	**Southeastern Taiwan**	**Mountainous Area**	**Pescadores Islands**	**Kinmen Islands**
Northern Taiwan									
Northwestern Taiwan	0.047								
Central Western Taiwan	0.000*	0.000*							
Southwestern Taiwan	0.000*	0.000*	0.447						
Northeastern Taiwan	0.019	0.405	0.000*	0.000*					
Southeastern Taiwan	0.431	0.109	0.000*	0.000*	0.04				
Mountainous Area	0.855	0.064	0.000*	0.000*	0.026	0.592			
Pescadores Islands	0.000*	0.000*	0.306	0.731	0.000*	0.000*	0.000*		
Kinmen Islands	0.000*	0.000*	0.000*	0.000*	0.000*	0.000*	0.000*	0.000*	
Matou Islands	0.000*	0.000*	0.000*	0.000*	0.000*	0.000*	0.000*	0.000*	0.086

* *p* < 0.01.

^$^ all the results was calculated by the Fisher’s exact test.

**Figure 3:**
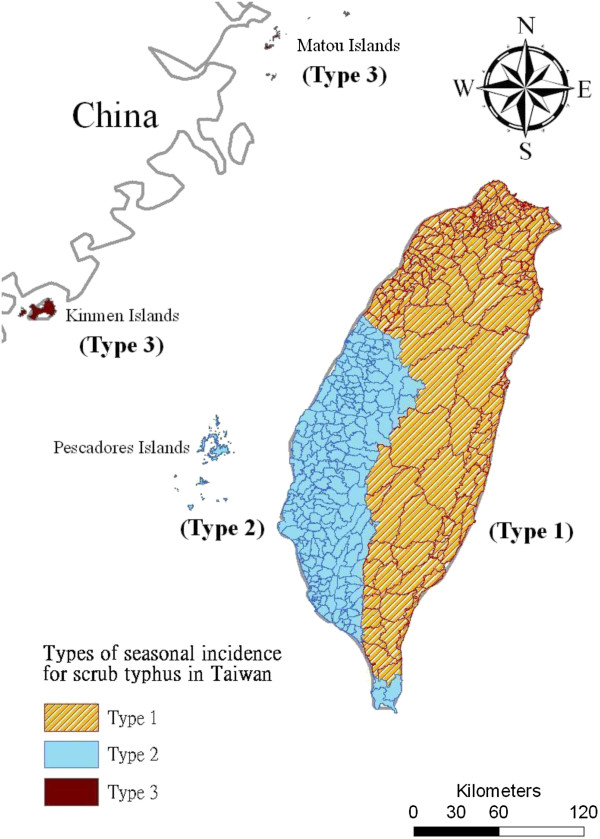
Types of seasonal incidence for scrub typhus in Taiwan.

Figure 3 shows a summary of the outcomes of matrix comparisons. The results are classified into the following 3 groups throughout the 10 local climate regions: (a) Type 1 comprises 5 local climate regions (the Northern, Northwestern, Northeastern, Southeastern, and mountainous area of Taiwan) that show no obvious seasonal variation; (b) Type 2 comprises 3 regions (Central Western and Southwestern Taiwan, and the Pescadore Islands) that show significantly seasonal variation, and have similar patterns of scrub typhus incidence; and (c) Type 3 comprises the Kinmen and Matou Islands, and shows a third pattern.

### Fitted results for the geographically weighted regression models

**Figure 4:**
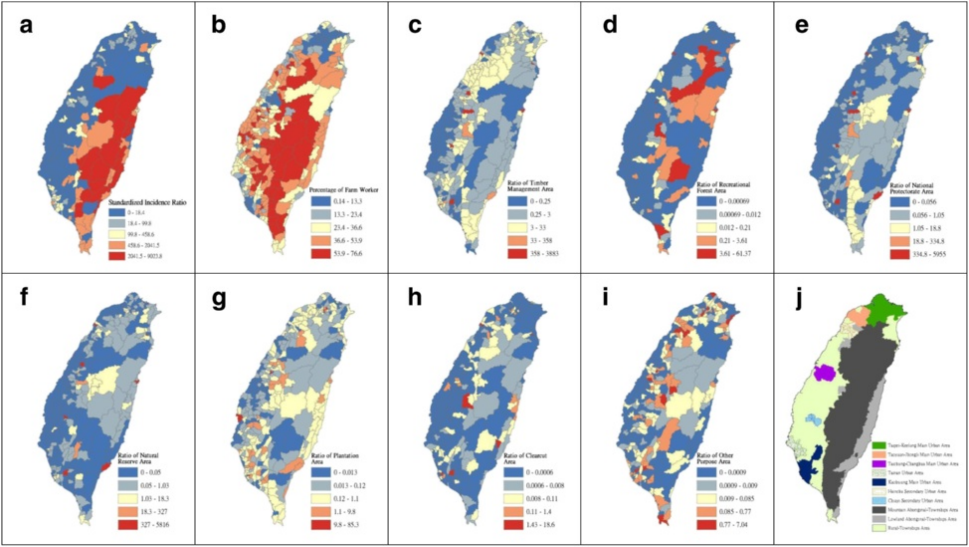
**Spatial maps of 349 townships in Taiwan in 2005.** (**a**) standardized incidence ratio (SIR) of scrub typhus, (**b**) percentage of farm workers, (**c**) land share ratio of timber management area, (**d**) land share ratio of recreational forest area, (**e**) land share ratio of national protective area, (**f**) land share ratio of natural reserve area, (**g**) land share ratio of plantation area, (**h**) land share ratio of clearcut area, (**i**) land share ratio of other purpose area, and (**j**) primary and secondary urban areas and rural and aboriginal townships in Taiwan.

**Figure 5:**
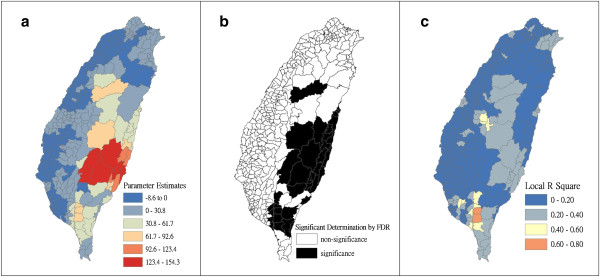
**The results of the GWR model for the standardized incidence ratio (SIR) of scrub typhus and the percentage of farm workers in 2005 on the main island of Taiwan: (a) parameter estimate, (b) significant determination by the false discovery rate (FDR), and (c) local R**^**2**^** value.**

**Figure 6:**
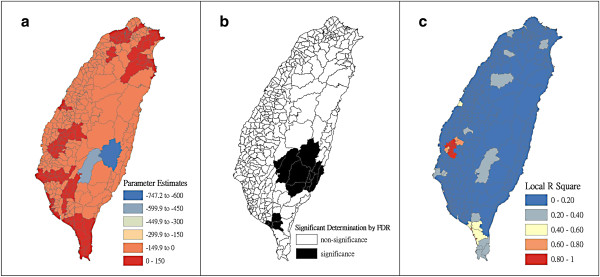
**The results of the GWR model for the standardized incidence ratio (SIR) of scrub typhus and the land share ratio of timber management area in 2005 on the main island of Taiwan: (a) parameter estimate, (b) significant determination by the false discovery rate (FDR), and (c) local R**^**2 **^**value.**

**Figure 7:**
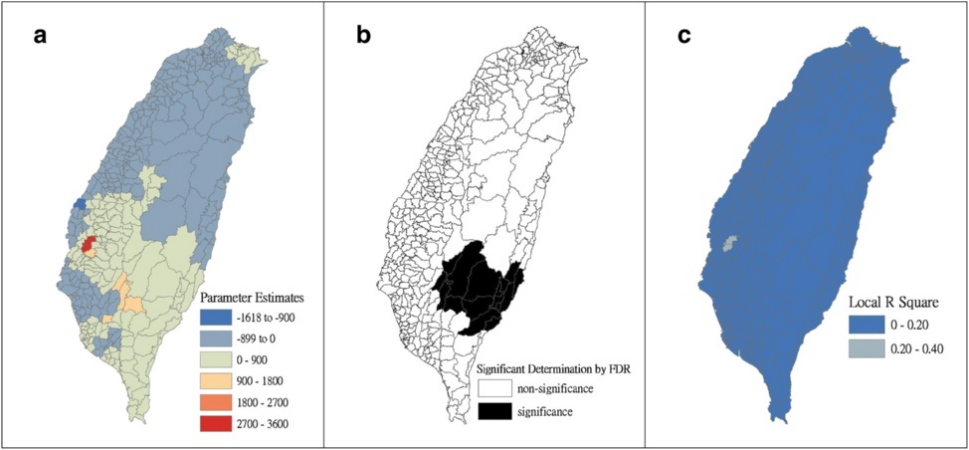
**The results of the GWR model for the standardized incidence ratio (SIR) of scrub typhus and the land share ratio of recreational forest area in 2005 on the main island of Taiwan: (a) parameter estimate, (b) significant determination by the false discovery rate (FDR), and (c) local R**^**2 **^**value.**

**Figure 8:**
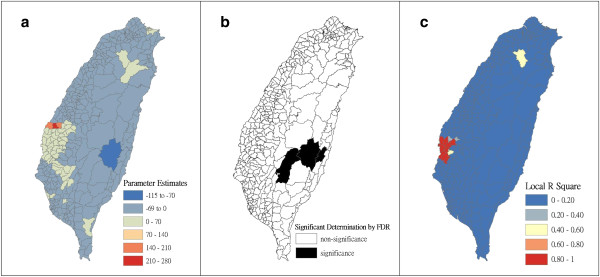
**The results of the GWR model for the standardized incidence ratio (SIR) of scrub typhus and the land share ratio of national protectorate area in 2005 on the main island of Taiwan: (a) parameter estimate, (b) significant determination by the false discovery rate (FDR), and (c) local R**^**2 **^**value.**

**Figure 9:**
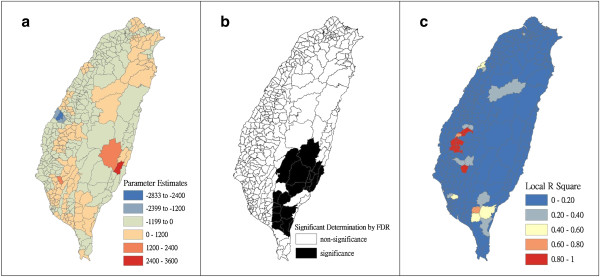
**The results of the GWR model for the standardized incidence ratio (SIR) of scrub typhus and the land share ratio of natural reserve area in 2005 on the main island of Taiwan: (a) parameter estimate, (b) significant determination by the false discovery rate (FDR), and (c) local R**^**2 **^**value.**

**Figure 10:**
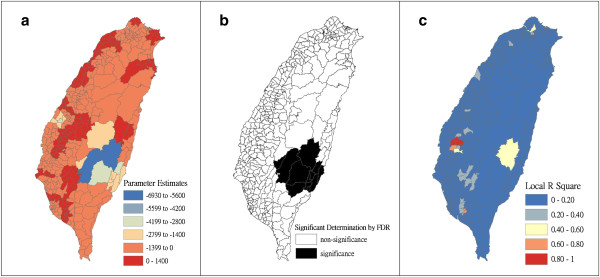
**The results of the GWR model for the standardized incidence ratio (SIR) of scrub typhus and the land share ratio of plantation area in 2005 on the main island of Taiwan: (a) parameter estimate, (b) significant determination by the false discovery rate (FDR), and (c) local R**^**2 **^**value.**

**Figure 11:**
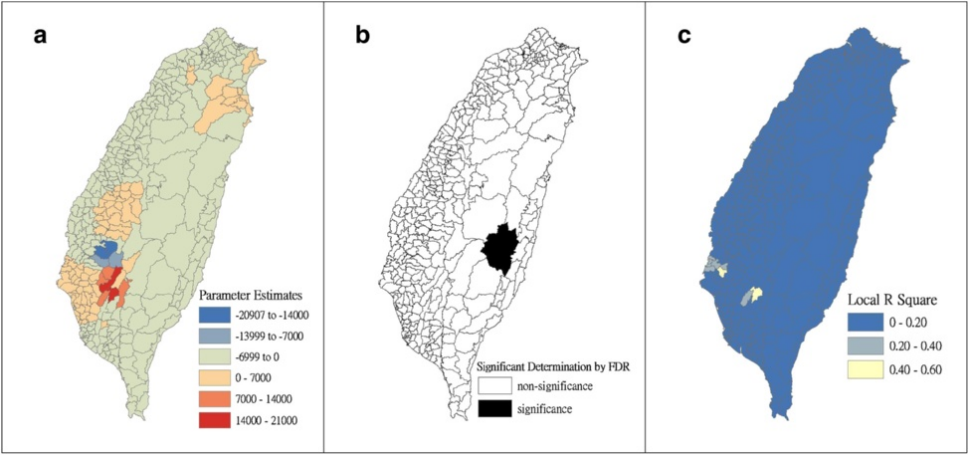
**The results of the GWR model for the standardized incidence ratio (SIR) of scrub typhus and the land share ratio of clearcut area in 2005 on the main island of Taiwan: (a) parameter estimate, (b) significant determination by the false discovery rate (FDR), and (c) local R**^**2 **^**value.**

**Figure 12:**
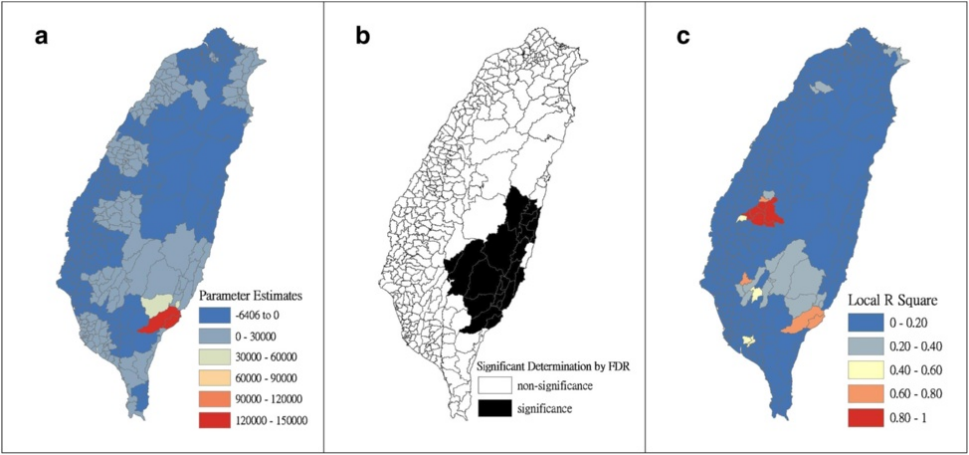
**The results of the GWR model for the standardized incidence ratio (SIR) of scrub typhus and the land share ratio of other purpose area in 2005 on the main island of Taiwan: (a) parameter estimate, (b) significant determination by the false discovery rate (FDR), and (c) local R**^**2 **^**value.**

Figure 4 shows a map of the geographical distribution of the SIR-district for scrub typhus, the percentage of farm workers, and the land share ratio (percentage) of forest-land use in Taiwan during 2005. We employed a survey to obtain data on forest-land usage based on the following categories: (a) timber management; (b) recreational forests; (c) national protectorates; (d) natural reserves; (e) plantations; (f) clear-cut areas; and (g) other purposes. In addition, we provided a map of administrative government areas in Taiwan for geographical reference (i.e., urban areas, rural townships areas, and aboriginal townships in lowland and mountainous areas), as shown in Figure 4j. Figures 5, 6, 7, 8, 9, 10, 11, 12 show the maps of the parameter estimates, the significant determination of the false discovery rate, and the explanatory power of the model (R^2^). In these maps, the figures for scrub typhus were fitted to the GWR models by using the following explanatory variables: (a) percentage of farm workers; (b) timber management area; (c) recreational forest area; (d) national protectorate area; (e) natural reserve area; (f) plantation area; (g) clear-cut area; and (h) other purpose area.

In the GWR models, the explanatory variable “farm workers” shows significantly positive parameter estimates in the following areas: (a) clusters of Southeastern Taiwan (low-land townships in Hualien and Taitung Counties); (b) central mountainous township areas and southern regions of Taiwan; and (c) Heping Township (Taichung County) (Figure 5). The explanatory variable “timber management area” shows significantly positive parameter estimates for Laiyi, Jiadong, Chunrih, and Fangliao Townships (all in Pingtung County), although the parameter estimates in Central Taiwan’s mountainous township region is negative (Figure 6). The explanatory variable “recreational forest area” shows significantly positive parameter estimates in the central mountainous township regions and Taitung County (Figure 7). The explanatory variable “natural reserve area” shows significantly positive parameter estimates in the southern mountainous township region and 5 townships (Jinfong, Daren, and Dawu Townships in Taitung County; Jhuosi and Fuli Townships in Hualien County), and negative parameter estimates in 8 townships (Taoyuan Township in Kaohsiung County; and Haiduan, Chenggong, Chihshang, Guanshan, Donghe, Luye, and Yanping Townships in Taitung County) (Figure 9). The explanatory variable “other purpose area” shows significantly positive parameter estimates in the central mountainous township region and Taitung County, and negative parameter estimates in 5 townships (Wanrong, Fonglin, Fongbin, Guangfu, and Rueisuei Townships in Hualien County) (Figure 12). The explanatory variables “national protectorate area,” “plantation area,” and “clear-cut area” show significantly negative parameter estimates, as shown in Figures 8, 10, and 11, respectively.

The R^2^ value represents the extent to which variations in a response variable can be determined by an explanatory variable. Therefore, the local R^2^ is considered the coefficient of the fitted results of the GWR models. The focus of geographical medical studies is to determine the spatial heterogeneity of specific health care events. Clusters are calculated using regression models, in which parameter estimates should have both significant and positive signs within the analyzed spatial units. The results of this study showed that the clusters are significantly associated with higher population densities of farm workers and specific forest-land uses (e.g., timber management, recreational forest, natural reserve, and other purpose use) in central and southern regions of the mountainous area, and in Southeastern Taiwan.

## Discussion

The results of this study showed that the Taiwan area’s scrub typhus incidence can be characterized into the following 3 types: (a) Type 1 is found in the northern, northwestern, northeastern, southeastern and the mountainous area of Taiwan. In this category, the incidence of scrub typhus is independent of the examined meteorological variables, and no obvious seasonal variations in incidence were observed. Thus, we hypothesized that the incidence of scrub typhus in Type 1 was unaffected by climatic conditions. (b) Type 2 applies to the central western and southwestern regions of Taiwan, as well as the Pescadore Islands, where the incidence of scrub typhus correlates positively with surface temperature. Furthermore, the incidence was considerably higher during the warm season. (c) Type 3 is representative of the Kinmen and Matou Islands, where the incidence of scrub typhus was significantly higher during the warm season and correlates positively with surface temperature and hours of sunshine.

The following species of trombiculidae are primarily responsible for transmitting scrub typhus in Taiwan: *Leptotrombidium deliense* and *L. scutellare* in the Kinmen Islands [24,25], *L. deliense* in the Pescadore Islands [24,26,27], and *L. imphalum* in Eastern Taiwan (Hualien and Taitung Counties) [28]. We tentatively conclude that *L. deliense* and *L. scutellare* cause a high incidence of scrub typhus in the Kinmen Islands, where temperature, precipitation, relative humidity, and the hours of sunshine correlate positively with incidence during the summer. In the Pescadore Islands, the proliferation of *L. deliense* is potentially related to higher temperatures. For *L. imphalum* in Eastern Taiwan (Hualien and Taitung Counties), no climatic effect was observed influencing the disease incidence.

Previous studies have shown that the geographical profile for OT Hyashi density shows that seropositive outcomes have been observed in small captured rodents and attached trombiculidae throughout Taiwan [14,28]. In this study, OT was clustered in less-developed areas with relatively low population densities, namely the mountainous area and Southeastern Taiwan. Previous studies have reported a higher incidence of scrub typhus in these areas [14,28]. Frequent human visitation to endemic areas is a critical factor that increases the probability of scrub typhus infection, and such visits might also provide additional food resources for small rodents. These factors potentially enhance the prevalence rate of scrub typhus. Thus, forest lands that are visited frequently are more likely to be associated with an increased prevalence of scrub typhus infection. The type of forest-land use partly determines the frequency of people’s visitations; for example, lands used for timber management, recreational forests, natural reserves, and other purposes are expected to be visited more frequently than national protectorates, plantations, and clear-cut areas. The analysis results showed significantly positive clusters associated with 4 forest-land use categories (timber management, recreational forest, natural reserve, and other purpose areas). In addition, farm workers in endemic areas increase the probability of contact with the OT-carrying trombiculidae, leading to higher infection rates. Numerous previous studies conducted in Japan [29], China [30], and Taiwan [14] have supported these findings.

## Conclusion

The findings of this study show that scrub typhus endemic regions in the Taiwan area are infested with “island type” scrub typhus. Type 1 shows no climatic effect. The incidence of scrub typhus in Type 2 correlates positively with high temperatures during the warm season. In Type 3, the peak occurrence of scrub typhus infection correlates with surface temperature and hours of sunshine. Higher SIR-district scrub typhus, which occurs in the central and southern portions of the mountainous area and Southeastern Taiwan, is associated with farm worker population density and the type of forest-land use (e.g., timber management, recreational forest, natural reserve, or other purposes). This study provides useful information for assessing spatial risk factors. The findings of this study can be used to improve the planning of health care policies in Taiwan and the implementation of effective health care services.

## Competing interests

The authors declare that they have no competing interests.

## Authors’ contributions

PJ was responsible for the study design, epidemiological enquiry, data collection, statistical calculations, and drafting of the manuscript. HC assisted with the study design and collection of the meteorological data. Both authors read and approved the final manuscript.

## Pre-publication history

The pre-publication history for this paper can be accessed here:

http://www.biomedcentral.com/1471-2334/13/191/prepub
